# Development of a certified reference material for the determination of polycyclic aromatic hydrocarbons (PAHs) in rubber toy

**DOI:** 10.1007/s00216-021-03796-5

**Published:** 2021-11-30

**Authors:** Thomas Sommerfeld, Christian Jung, Juliane Riedel, Tatjana Mauch, Andreas Sauer, Matthias Koch

**Affiliations:** Bundesanstalt für Materialforschung und -prüfung (BAM), Richard-Willstätter-Straße 11, 12489 Berlin, Germany

**Keywords:** PAHs, Consumer products, Toys, Chemical safety, Certified reference material, Quality assurance

## Abstract

Polycyclic aromatic hydrocarbons (PAHs) are a large group of priority organic pollutants, which contaminate environmental compartments, food, and consumer products as well. Due to their frequent occurrence associated with elevated levels of PAHs, plastic and rubber parts of consumer products and toys are particular sources of exposure. Although European maximum levels exist for eight carcinogenic PAHs in consumer products and toys according to REACH Regulation (EC) No. 1907/2006, certified reference materials (CRM) are still not available. To overcome this lack, the first CRM for the determination of PAHs in rubber toys (BAM-B001) was developed according to the requirements of ISO Guide 35. The whole process of CRM development including preparation, homogeneity and stability studies, and value assignment is presented. The assignment of the certified mass fractions was based upon in-house study at BAM using stable isotope dilution analysis (SIDA) gas chromatography mass spectrometry (GC–MS). The obtained values were confirmed by the results of two interlaboratory comparison (ILC) studies with more than 50 expert laboratories from Germany and China. The mass fractions of 14 PAHs including all REACH and GS mark regulated compounds were certified ranging between 0.2 and 15.4 mg/kg accompanied by expanded uncertainties (coverage factor *k* = 2). In addition, informative values were determined for 4 PAHs, mainly due to higher uncertainties and/or lack of ILC data for confirmation. BAM-B001 is intended for analytical quality control particularly based on the AfPS GS 2019:01 PAK method and contributes to improve the chemical safety of consumer products including toys.

**Graphical abstract:**

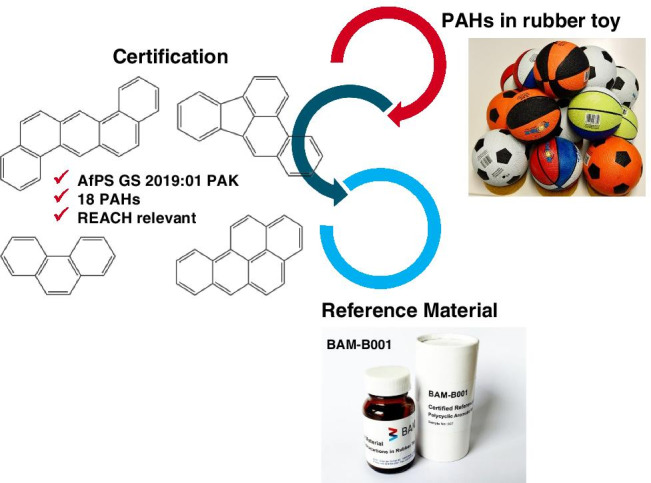

**Supplementary Information:**

The online version contains supplementary material available at 10.1007/s00216-021-03796-5.

## Introduction

The sustainability of consumer goods including life cycle assessment and product safety including consumers’ health has gained increased awareness and importance. In 2017, the European Union (EU) imported toys worth almost 7.4 billion € with Asia being the biggest supplier, especially China accounting for 86% of toy imports [1]. According to the Rapid Alert System for dangerous non-food products (RAPEX), most alerts in 2019 were issued within the toy product category, with chemical risks to health identified as the second highest cause for notification. Besides phthalates, polycyclic aromatic hydrocarbons (PAHs) were among the chemicals frequently detected in toys [2]. Monitoring the chemical composition of toys is therefore an important prerequisite in ensuring toy safety.

**Fig. 1:**
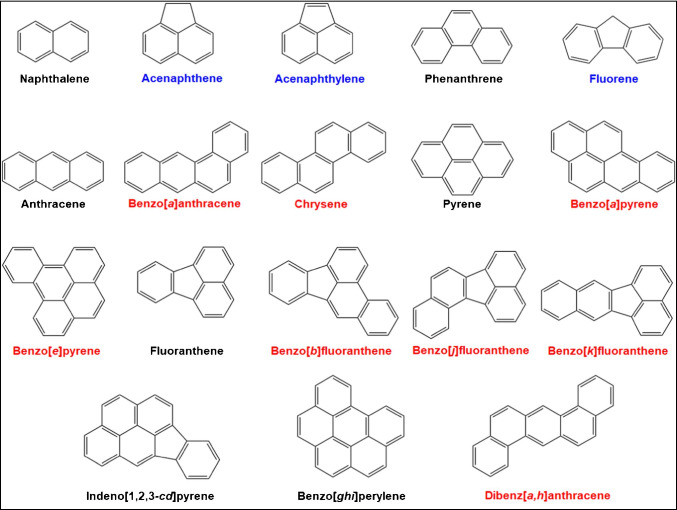
Polycyclic aromatic hydrocarbons (PAHs) to be controlled in consumer products including toys. *Red*: 8 PAHs regulated acc. to REACH; *red* + *black*: 15 PAHs regulated acc. to current AfPS/German GS mark certification (2019); *red* + *black* + *blue*: 18 PAHs regulated acc. to former AfPS/German GS mark certification (2014)

PAHs are a ubiquitous group of several hundred chemically related compounds, formed both during biological processes and as products of incomplete combustion [3]. These persistent compounds belong to the priority organic pollutants in environmental compartments like water and soil and are found as contaminants in feed and food, e.g., in olive oils. However, PAHs can also be detected in a variety of consumer products including toys made from virgin or recycled rubber, especially in plastic and rubber parts. They are present as impurities in raw materials used for the production of such goods, particularly in extender oils and in carbon black [4, 5]. Exposure to PAHs poses a health risk because many congeners have toxic, mutagenic, and/or carcinogenic properties [3, 6, 7]. They reach humans via different routes, with dermal contact or inhalation being the primary routes of exposure [8]. Toddlers and young children constitute a high-risk group due to their mouthing behavior which allows PAHs to enter their body much more easily. Thus, the mass fractions of eight priority PAHs—all presumed carcinogens (category 1B according to European classification)—were restricted to 0.5 mg/kg for toy and childcare products and to 1 mg/kg for all other consumer products. The corresponding Commission Regulation (EU) 1272/2013 amending Annex XVII to the REACH Regulation 1907/2006 [9] refers to benz[*a*]anthracene, chrysene, benzo[*b*]fluoranthene, benzo[*k*]fluoranthene, benzo[*j*]fluoranthene, benzo[*e*]pyrene, benzo[*a*]pyrene and dibenz[*a,h*]anthracene (Fig. 1).

To ensure the chemical safety of consumer products, several methods have been developed based on total extraction [10] and migration of PAHs from products [5]. In Germany, standard method AfPS GS 2019:01 PAK [11] was developed and validated to control 15 PAHs in consumer/toy products based on gas chromatography mass spectrometry (GC–MS). In addition to the eight REACH-regulated compounds, naphthalene, phenanthrene, anthracene, pyrene, fluoranthene, indeno[1‚2,3-*cd*]pyrene, and benzo[*ghi*]perylene are included in the PAH-15 list (Fig. 1). Since July 2020, this list is applied for PAH testing of consumer products for GS mark certification (GS: Tested Safety) according to the German Product Safety Law replacing the former PAH-18 list of AfPS which addressed also acenaphthylene, acenaphthene, and fluorene (Fig. 1).

Although maximum levels for PAHs are in force, there are still no certified reference materials (CRM) for the analysis of PAHs in rubber toys. Reference materials (RM) and especially CRM are required to verify the accuracy of analytical measurements. Thus, the goal of the presented project was to develop the first CRM for PAHs in rubber toys (BAM-B001). Because matrix materials spiked/fortified with the respective analytes are generally to be avoided whenever possible, the aim was to produce a real-life material as CRM, representative for a toy product with PAH contents in the range of the REACH limits and real congener pattern. To achieve the broadest possible applicability, the “PAH-18 list” formed the basis of CRM development.

Certification of BAM-B001 was planned by an in-house study at BAM with two independent workplaces (three data sets) taking into account ISO 17034 [12] and the relevant ISO-Guides [13, 14]. Data from two independent interlaboratory comparison (ILCs) studies involving more than 50 laboratories with documented experience in PAH analysis of consumer products were used to confirm the in-house certification approach. BAM-B001 is intended to be used for performance control and validation of analytical methods for the determination of PAHs in rubber toys and similar consumer products [15].

## Materials and methods

### Material preparation

In a survey of different plastic and rubber toys, a certain type of rubber ball was identified and selected as suitable starting material due to its mainly relevant PAH contents [15]. A total amount of about 15 kg was procured in various toy stores in Berlin/Germany.

The following procedure was applied to prepare the candidate reference material: manually cutting each ball into pieces (2 × 2 cm), cryo-milling (liquid nitrogen) to < 8 mm using a cutting mill, cryo-milling (liquid nitrogen) to < 2 mm using a centrifugal mill, sieving and collecting the < 2-mm fraction (2-mm sieve) for fabric removal (> 2 mm), cryo-milling (liquid nitrogen) to < 500 µm using a centrifugal mill, sieving and collecting the < 500-µm fraction (500-µm sieve) for fabric removal, and homogenization of the < 500-µm sieve fraction for 18 h using a drum hoop mixer.

After final processing, 9265 g of the candidate material was obtained. For the German/Chinese ILC (ILC-1), 115 amber glass bottles were manually filled with 12 g each. The remaining candidate material (7882 g) was used to prepare 778 units of BAM-B001, which were filled into 50-mL screw-capped amber glass bottles containing (10.1 ± 0.1) g and numbered in the order in which they left the manual filling process. All of the bottles were stored at − 20 °C immediately after filling and were kept in the freezer until dispatch from BAM.

### Analytical methods

Although high-performance liquid chromatography (HPLC) coupled with diode array detection (DAD) or fluorescence detection (FLD) and GC–MS are commonly used methods for routine analysis of PAH in different samples, GC–MS is prescribed for PAH determination in consumer products according to method AfPS GS 2019:01 PAK. Therefore, this method was applied for all characterizations of BAM-B001 as described below. To further improve the current AfPS GS 2019:01 PAK method which prescribes at least three isotopically labeled internal standards covering the range of PAHs’ molecular weights, each PAH congener of BAM-B001 was quantified using its deuterated counterpart for all characterization analyses [15].

Two independent workplaces (A and B) were provided for carrying out the measurements in the frame of the in-house study of BAM-B001. Each workplace included an operator performing specific sample preparation followed by sample analyses on their instrument with individually prepared PAH standards/calibration, and separate data evaluation. Analyses for material characterization (homogeneity, stability, and certification) were performed by operator A using method A and GC–MS system A. Operator B was exclusively involved in the certification measurements using method B and GC–MS system B.

#### Method A

Sample preparation was done according to method AfPS GS 2019:01 PAK. Each step of the sample preparation was performed in a gravimetrically controlled manner, even though volumes are given below. 0.5 g of the rubber toy sample was weighed into a 40-mL screw-capped glass vial. After addition of 20 mL of toluene, the sample was extracted by ultrasonication (PTIC-X-ES, Allpax, Papenburg, Germany) at 60 °C for 1 h. After the extract cooled to room temperature, an aliquot of 0.5 mL was transferred to a 1.5-mL HPLC vial. To this extract aliquot, 100 µL of a deuterated internal standard solution (ISTD) was added and subsequently analyzed by GC–MS system A (Table 1).

**Table 1:** Instrumental parameters of the two GC–MS systems A and B used for PAH analysis of BAM-B001

Instrumental parameter	*GC–MS system A*	*GC–MS system B*
GC system	6890 N (Agilent) + KAS 4 (Gerstel)	7890A (Agilent)
Column	Select PAH (Agilent)	DB-17 ms (Agilent)
Column dimensions	30 m × 0.25 mm × 0.15 µm ID	60 m × 0.25 mm × 0.25 µm ID
Oven program	70 °C (1 min) → (85 °C/min) 180 °C → (3 °C/min) 230 °C (7 min) → (28 °C/min) 280 °C (10 min) → (14 °C/min) 350 °C (3 min)	80 °C (1 min) → (5 °C /min) 320 °C (33 min)
Carrier gas	He 5.0 (2 mL/min)	He 5.0 (2 mL/min)
Injection	5 µL (large volume injection, LVI)	1 µL (splitless at 320 °C)
MS system	MSD 5975B inert XL (Agilent)	MSD 5975C inert XL (Agilent)
Ionization	70 eV (EI)	70 eV (EI)
Acquisition mode	SIM	SIM

#### Method B

The same extraction procedure as for method A (AfPS) was performed. A sample amount of 0.5 g was weighed into a 22-mL screw-capped glass vial. After addition of 30 µL of deuterated ISTD solution and 20 mL of toluene, the sample was extracted by ultrasonication (Bandelin, Berlin, Germany) at 60 °C for 1 h. Solid particles were separated from the extract by filtration (folded filters 595 ½, Schleicher & Schuell). The glass vial and filter were rinsed with 3 × 5 mL of toluene and combined with the extract. Subsequent sample preparation steps followed a method successfully applied in the CCQM-K146 study [16]: The total extract was evaporated to near dryness in a Kuderna-Danish flask at 50 °C in a nitrogen stream. The residue was redissolved in 1 mL of cyclohexane/ethyl acetate (1:1) and transferred to a 5-mL flask. After rinsing the Kuderna-Danish flask with 3 × 1 mL of cyclohexane/ethyl acetate (1:1) in the 5-mL flask and filling the flask to the mark, the turbid extract was filtered using a 0.22 µm PTFE filter (Macherey–Nagel, Düren, Germany). A volume of 4 mL of the clear extract was injected into a GPC system (LCTech, Obertaufkirchen, Germany) equipped with a 660 × 40 mm column (ID = 25 mm) filled with Bio-Beads S-X3, 200–400. The selected PAH fraction was transferred to a Kuderna-Danish flask and evaporated at 50 °C and 300 mbar almost to dryness. Subsequent evaporation to dryness was completed in a gentle stream of nitrogen. The yellow residue was redissolved in 1 mL of petroleum ether and purified by applying this solution to a 6-mL SPE cartridge filled with 2 g alumina deactivated with 11% water and covered with 0.5 g anhydrous sodium sulfate (Merck, Darmstadt, Germany). The Kuderna-Danish flask was rinsed with 3 × 5 mL of petroleum ether, which was also applied to the SPE cartridge. The PAH fraction was eluted by adding 10 mL of petroleum ether to the SPE cartridge. After adding 1 mL of toluene to the petroleum ether eluate, the solution was evaporated at room temperature almost to dryness and adjusted to 1 mL with toluene. The prepared sample was transferred to a GC vial and analyzed using GC–MS system B (Table 1).

#### GC–MS analysis

For better performance (increased sensitivity, reduced background), all 18 native PAH analytes and 16 deuterated ISTD were analyzed by GC–MS in single ion monitoring (SIM) mode (Table 2). PAHs were quantified using stable isotope dilution analysis (SIDA) mass spectrometry which is recognized as a primary ratio method of measurement by the Consultative Committee for Amount of Substance (CCQM). Almost all PAHs could be quantified using the corresponding deuterated congener as ISTD. Where not possible (benzo[*j*]fluoranthene and benzo[*e*]pyrene), the alternatively used ISTDs are indicated in Table 2.

**Table 2:** Measurement parameters for PAH determination of BAM-B001 using the GC–MS systems A and B

Compound	Sum formula	SIM (m/z)	Retention time (min)		Quantified by using internal standard
			*GC–MS A*	*GC–MS B*	
Naphthalene	C_10_H_8_	128.1	2.97	14.52	D_8_-Naphthalene
Acenaphthylene	C_12_H_8_	152.1	4.11	22.56	D_8_-Acenaphthylene
Acenaphthene	C_12_H_10_	*)	4.23	23.18	D_10_-Acenaphthene
Fluorene	C_13_H_10_	166.1	5.01	25.61	D_10_-Fluorene
Phenanthrene	C_14_H_10_	178.1	7.73	30.99	D_10_-Phenanthrene
Anthracene	C_14_H_10_	178.1	7.88	31.14	D_10_-Anthracene
Fluoranthene	C_16_H_10_	202.1	13.41	37.16	D_10_-Fluoranthene
Pyrene	C_16_H_10_	202.1	15.01	38.61	D_10_-Pyrene
Benz[*a*]anthracene	C_18_H_12_	228.1	24.56	44.54	D_12_-Benz[*a*]anthracene
Chrysene	C_18_H_12_	228.1	25.41	44.91	D_12_-Chrysene
Benzo[*b*]fluoranthene	C_20_H_12_	252.1	30.60	49.81	D_12_-Benzo[*b*]fluoranthene
Benzo[*k*]fluoranthene	C_20_H_12_	252.1	30.71	49.93	D_12_-Benzo[*k*]fluoranthene
Benzo[*j*]fluoranthene	C_20_H_12_	252.1	30.78	51.00	D_12_-Benzo[*k*]fluoranthene
Benzo[*e*]pyrene	C_20_H_12_	252.1	32.37	52.72	D_12_-Benzo[*a*]pyrene
Benzo[*a*]pyrene	C_20_H_12_	252.1	32.68	52.01	D_12_-Benzo[*a*]pyrene
Indeno[1,2,3-*cd*]pyrene	C_22_H_12_	276.1	39.84	62.35	D_12_-Indeno[1,2,3-*cd*]pyrene
Dibenz[*a,h*]anthracene	C_22_H_14_	278.1	39.93	59.30	D_14_-Dibenz[*a,h*]anthracene
Benzo[*ghi*]perylene	C_22_H_12_	276.1	41.00	62.35	D_12_-Benzo[*ghi*]perylene
D_8_-Naphthalene	C_10_D_8_	136.1	2.95	14.42	–
D_8_-Acenaphthylene	C_12_D_8_	160.1	4.10	22.48	–
D_10_-Acenaphthene	C_12_D_10_	164.2	4.22	23.00	–
D_10_-Fluorene	C_13_D_10_	176.2	4.96	25.43	–
D_10_-Phenanthrene	C_14_D_10_	188.2	7.64	30.85	–
D_10_-Anthracene	C_14_D_10_	188.2	7.80	31.03	–
D_10_-Fluoranthene	C_16_D_10_	212.2	13.29	37.05	–
D_10_-Pyrene	C_16_D_10_	212.2	14.89	38.51	–
D_12_-Benz[*a*]anthracene	C_18_D_12_	240.2	34.27	44.41	–
D_12_-Chrysene	C_18_D_12_	240.2	25.06	44.81	–
D_12_-Benzo[*b*]fluoranthene	C_20_D_12_	264.2	30.47	49.69	–
D_12_-Benzo[*k*]fluoranthene	C_20_D_12_	264.2	30.60	49.81	–
D_12_-Benzo[*a*]pyrene	C_20_D_12_	264.2	32.52	51.86	–
D_12_-Indeno[1,2,3-*cd*]pyrene	C_22_D_12_	288.2	39.73	59.10	–
D_14_-Dibenz[*a,h*]anthracene	C_22_D_14_	292.2	39.75	58.98	–
D_12_-Benzo[*ghi*]perylene	C_22_D_12_	288.2	40.91	62.06	–

^*^) m/z = 153 (*GC–MS system A*); m/z = 154 (*GC–MS system B*)

The certified standard SRM 2260a (NIST, Gaithersburg, USA) containing all relevant PAHs was used for PAH calibration for both workplaces. The deuterated internal standards were provided via the PAH-Mix 9 (Dr. Ehrenstorfer GmbH, Augsburg, Germany). Nine-point (*GC–MS system A*) and twelve-point (*GC–MS system B*) calibrations were used for quantification of the measured area ratios. Based on regression analysis, the calibration functions of all PAHs were assumed to be linear.

### Homogeneity study

The homogeneity study assesses the distribution of the analyte in all the units bottled. That means that an uncertainty contribution resulting from possible heterogeneity (between-unit inhomogeneity) has to be quantified. An estimate for the between-bottle inhomogeneity of the candidate CRM was assessed by determining PAH in 12 units equidistantly selected from the produced batch of the 778 units. The selected units were processed four times each according to the analytical method A described above. All units were extracted under repeatability conditions in one day (12 × 4 = 48 extractions). The extracts were analyzed in randomized manner under repeatability conditions in such a way that all 48 extracts were quantified against one calibration [15].

The estimate of analyte-specific (in)homogeneity contribution *u*_bb_ to be included in the overall uncertainty budget of BAM-B001 was calculated according to ISO Guide 35 [14] using Eq. 1:

1 $${{u}_{\mathrm{bb}}=\sqrt{\frac{{\text{MS}}_{\text{between}}- {MS}_{\mathrm{within}}}{\text{n}}}}$$

*MS*_between_: mean of squared deviation between units (from one-factorial ANOVA)
*MS*_within_: mean of squared deviation within units (from one-factorial ANOVA)
*n*: number of replicate determinations per unit (*n* = 4)

### Stability study

Experience with temperature-induced deterioration of PAHs is available for various environmental materials when the PAHs are mainly adsorbed on the particle surface (e.g., soils, sediment, wood). Much less experience exists for materials such as rubber where PAHs are embedded. Therefore, selected units of the candidate material were subjected to an isochronous accelerated aging stability study [17] at temperatures ranging from + 4 to + 60 °C over 12 months. At the end of the respective periods, the individual units were stored at − 20 °C. All units were analyzed for PAHs according to method A described above under repeatability conditions together with reference samples which had been kept at − 20 °C since bottling [15].

For the evaluation of stability data, there is a kinetic approach [18] that has been used successfully in the past for a variety of organic compounds in food and environmental matrices: From semi-logarithmic plots of measured values versus time, effective deterioration rates *k*_eff_(*T*) were tested against an *Arrhenius* model describing the temperature dependence of the deterioration rates. This approach should also be used for BAM-B001. Further investigations of the stability of BAM-B001 are planned as part of the post-certification monitoring.

### Characterization and value assignment

The determination of the certified PAH mass fractions of BAM-B001 was based on the in-house study at BAM, in which the candidate material was analyzed at two independent workplaces (A and B) using the SIDA GC–MS method. As an independent third data set, the results from the homogeneity study were included using separate materials, standards, and calibration, performed several weeks apart from in-house certification [15].

For in-house certification, 5 units of the candidate reference material were selected and treated according to the analytical methods A and B described above. Five independent replicates were analyzed for each unit, resulting in 25 analyses at each workplace and 50 results for each PAH congener in total. Additionally, 48 results for each PAH congener were obtained from the homogeneity study as the third data set, so a total of 98 independent results were obtained for each PAH congener.

Two ILCs with experienced laboratories could be used to support and to confirm BAM’s in-house certification study of BAM-B001. ILC-1 was conducted for PAHs in rubber toys in the framework of the Sino-German working group “Product safety” under the responsibility of the Federal Ministry for Economic Affairs and Energy (BMWi). BAM organized ILC-1 with > 50 expert laboratories from China and Germany focusing on the eight REACH-regulated PAH congeners and two additional PAHs: indeno[1,2,3-*cd*]pyrene and benzo[*ghi*]perylene. ILC-2 was conducted as part of the method validation study for PAHs in consumer products, which led to the adoption of the standard procedure [19]. The subject of ILC-2 was the analysis of all 18 PAHs that are also relevant to BAM-B001.

## Results and discussion

The certification campaign of BAM-B001 implied homogeneity evaluation, stability testing, in-house characterization for assignment of the certified value including two supporting ILCs, and calculation of the uncertainty budget also enabling a statement of traceability.

### Assessment of homogeneity

**Fig. 2:**
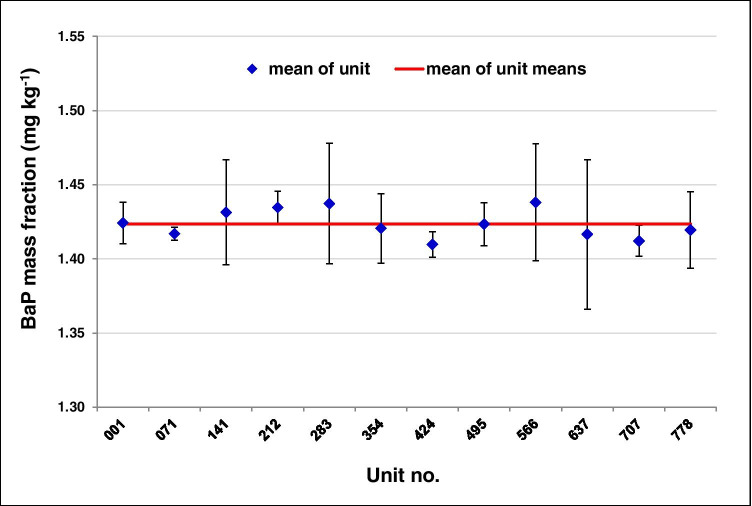
Homogeneity study of BAM-B001, example benzo[*a*]pyrene (BaP): mean values of BaP for 12 selected units with their corresponding standard deviations (*n* = 4) represented by error bars. The mean of all unit means (red line) is 1.424 mg kg^−1^

Based upon the inherent distribution of PAH in the rubber material, milling, sieving, and thorough batch homogenization, a satisfactory level of homogeneity was expected for BAM-B001. The results of the homogeneity study, displayed in Fig. 2 for benzo[*a*]pyrene (BaP) as an example, were visually inspected for trends and outliers. They show no trend with respect to the measurement and filling order, and also no outlier was identified. The results were evaluated using one-factorial ANOVA and displayed in Table S1 (Supplementary Information, ESM) together with the contributions due to between-bottle homogeneity (*u*_bb_) according to Eq. 1.

In the case of *MS*_between_ < *MS*_within_, Eq. 1 is not applicable, and the uncertainty contribution is set to zero according to ISO Guide 35 as done for several PAHs (Table S1, ESM). In principle, there could be three explanations for this effect (*F*-value < 1): (*i*) a very homogenous distribution of PAHs between bottles; (*ii*) a large inhomogeneity of PAHs within the bottles (for 0.5 g sample amount), and/or (*iii*) a high measurement uncertainty. The latter is rather unlikely due to the use of the SIDA GC–MS method. Increased inhomogeneity within the bottle caused by a small sample size cannot be completely excluded. On the other hand, a satisfactory repeatability (1.3–3.4%) was found for PAH contents in the range of 2 mg/kg (fluorene, anthracene, benz[*a*]anthracene, chrysene). It should be taken into account that according to the AfPS GS 2019:01 PAK method, a sample amount of 0.5 g is prescribed and further grinding of the particles lower than 500 µm was not possible for technical reasons.

The homogeneity test results obtained, valid for a sample size of 0.5 g (= minimum sample intake), indicate suitability for the intended use. Test portions below this minimum sample intake should not be used to avoid possible inhomogeneities.

### Assessment of stability

The stability data evaluation based upon a kinetic approach (*Arrhenius* model) [18]—successfully applied for several previous CRM projects—could not be used for BAM-B001. This model failed because PAH recoveries of approximately 100% were obtained after aging over 12 months even for higher storage temperatures (Table S2, ESM).

The stability tests show that storage temperatures of − 20 °C, + 4 °C, and even room temperature are sufficient for a minimum shelf-life target of 5 years. Therefore, the uncertainty contribution due to long-term stability was set to zero (see also section “Measurement uncertainty”). Exposure to temperatures higher than room temperature may shorten the validity period of BAM-B001, especially for low molecular weight PAHs. Hence, an expiry date of 2 years after delivery is specified, provided that the sample is stored at a temperature of + 4 °C or below at the user’s premises. The stability of the CRM is not affected by short-term handling at ambient temperature during transport and use. Therefore, BAM-B001 can be shipped at room temperature.

### Characterization and value assignment

The outcome of the in-house certification study (Table 3) results from three data sets (two independent workplaces using their individual methods A and B and the homogeneity study as third data set), representing a total of 98 results for each PAH congener. The individual results of data sets are summarized in Table S3 (ESM).

**Table 3:** Results of the in-house certification study of BAM-B001

Compound	*x*_char_^a^ (mg kg^−1^)	SD^b^ (mg kg^−1^)	*u*_char_^c^ (mg kg^−1^)
Naphthalene	0.0921	0.0674	0.0389
Acenaphthylene	1.6364	1.3232	0.7640
Acenaphthene	0.6341	0.2392	0.1381
Fluorene	1.7143	0.1486	0.0858
Phenanthrene	15.3863	0.5737	0.3312
Anthracene	2.8938	0.8755	0.5055
Fluoranthene	4.2735	0.2621	0.1513
Pyrene	11.3551	0.6535	0.3773
Benz[*a*]anthracene	2.1740	0.1478	0.0853
Chrysene	2.0808	0.0328	0.0189
Benzo[*b*]fluoranthene	0.5739	0.0230	0.0133
Benzo[*k*]fluoranthene	0.2133	0.0147	0.0085
Benzo[*j*]fluoranthene	0.3999	0.0151	0.0087
Benzo[*e*]pyrene	1.2078	0.1182	0.0682
Benzo[*a*]pyrene	1.4056	0.0307	0.0178
Indeno[1,2,3-*cd*]pyrene	0.2834	0.0463	0.0267
Dibenz[*a,h*]anthracene	0.1181	0.0154	0.0089
Benzo[*ghi*]perylene	1.4348	0.0195	0.0113

^a^Mean of three data set means

^b^Standard deviation of three data set means

^c^Standard uncertainty of the mean of data set means, i.e., SD/$\sqrt{N}$ (*N* = number of data sets = 3)

### Interlaboratory comparison studies

Each ILC participant was given one bottle of BAM-B001 and requested to analyze the bottle in triplicate according to AfPS [11] for ILC-1, or in duplicate according to the similar method [19] for ILC-2. The results of both ILCs are displayed in Table 4.

**Table 4:** Results of the interlaboratory comparison studies ILC-1 and ILC-2 for BAM-B001

Compound	ILC-1			ILC-2		
	Mean^a^	SD_ILC_^c^	*N*^d^	Mean^b^	SD_ILC_^c^	*N*^d^
Naphthalene						
Acenaphthylene				1.856	0.313	15
Acenaphthene				0.472	0.076	14
Fluorene				1.452	0.066	12
Phenanthrene				15.541	1.324	14
Anthracene				3.035	0.224	15
Fluoranthene				4.492	0.162	15
Pyrene				11.401	0.384	14
Benz[*a*]anthracene	2.455	0.156	36	2.871	0.149	16
Chrysene	2.488	0.175	36	2.645	0.179	16
Benzo[*b*]fluoranthene	0.674	0.061	35	0.649	0.042	16
Benzo[*k*]fluoranthene	0.288	0.037	34	0.248	0.021	12
Benzo[*j*]fluoranthene	0.429	0.042	34	0.373	0.023	14
Benzo[*e*]pyrene	1.220	0.082	36	1.135	0.041	16
Benzo[*a*]pyrene	1.433	0.097	35	1.364	0.071	16
Indeno[1,2,3-*cd*]pyrene	0.300	0.033	30	0.331	0.033	16
Dibenz[*a,h*]anthracene	0.205	0.040	18			
Benzo[*ghi*]perylene	1.254	0.094	31	1.250	0.071	14

^a^Robust mean (*Hampel* estimator)

^b^Arithmetic mean after outlier elimination

^c^Standard deviation of the ILC

^d^Number of data sets with quantitative results after outlier elimination

Robust statistics were applied to evaluate the results of ILC-1. The *Hampel* estimator was used for the robust mean, and the Q-method was applied to determine the robust standard deviation of ILC-1 according to ISO 13528 [20], both calculated by means of ProLab plus software (Quodata, Dresden, Germany). The PAH congeners indicated with crossed fields in Table 4 for ILC-1 were the not subject of the study. The results of ILC-2 were statistically evaluated by means of ProLab plus software, using the statistical approaches according to ISO 5725–2 [21]. The two PAH congeners indicated with crossed fields in Table 4 for ILC-2 could not be statistically evaluated due to a proportion of higher than 25% for results “ < LOQ” (LOQ: limit of quantification).

The comparability of ILC results with in-house certification results was tested using the (amended) ${E}_{n}$ criterion on the difference between the ILC mean ${x}_{1}$ and the BAM in-house value ${x}_{2}$ according to Eq. 2:

2 $${E}_{n}= \frac{\left|{x}_{1}-{x}_{2}\right|}{2\sqrt{{SD}_{\mathrm{ILC}}^{2}+{u}_{\mathrm{char}}^{2}}}$$

${SD}_{ILC}$standard deviation of the ILC.

${u}_{char}$uncertainty of characterization (in-house study).

The factor 2 in Eq. 2 converts the standard uncertainties in the denominator into expanded uncertainties. The resulting ${E}_{n}$ values were 0.1 to 1.2 for ILC-1 and 0.1 to 1.6 for ILC-2 with one exception slightly exceeding the critical value of ${E}_{n}$=2: benz[*a*]anthracene, ${E}_{n}$ 2.03 (ILC-2) but ${E}_{n}$ 0.8 for ILC-1. Thus, the results of the ${E}_{n}$ criterion indicate that the outcomes of ILCs are in good agreement with the in-house certification results based on the SIDA GC–MS at BAM.

### Measurement uncertainty

The combined uncertainty *u*_com_ of the candidate reference material was calculated according to Eq. 3.

3 $${u}_{\mathrm{com}}={x}_{\mathrm{char}}\bullet \sqrt{{u}_{\mathrm{char},r}^{2}+{u}_{\mathrm{bb},r}^{2}+{u}_{\mathrm{pur},r}^{2}+{u}_{\mathrm{hand},r}^{2}+{u}_{\mathrm{lts},r}^{2}}$$

*x*_*char*_certified value (mean of operator means).

${u}_{char,r}$uncertainty of characterization.

${u}_{bb,r}$contribution due to the between-bottle inhomogeneity as recommended in [14, 22]

${u}_{pur,r}$uncertainty of the native PAH calibration standard SRM 2260a (NIST).

${u}_{hand,r}$contribution from sample handling (pragmatic approach: 3% for all PAH congeners).

${u}_{lts,r}$contribution from long-term stability (sufficiently stable for shelf-life of 5 years, no uncertainty contribution required).

While *u*_char_, *u*_bb_, and *u*_pur_ are resulting from measurable contributions, the uncertainty from handling *u*_hand_ is a combined, rather worst-case estimate for all gravimetric and volumetric sample-handling procedures including the calibration uncertainty. Table 5 displays the data to calculate the combined uncertainty *u*_com_ for phenanthrene as a representative example (three more PAHs are displayed in Tables S4–S6). The calculated *u*_com_ values for all PAHs are summarized in Table S7. The terms *u*_char_ and *u*_hand_ were found to be the dominant uncertainty contributions to a similar extent for all PAHs.

**Table 5:** Uncertainty contributions for the calculation of the combined uncertainty (*u*_com_) of phenanthrene in BAM-B001

Parameter	Characterization	Homogeneity	Purity	Handling	Stability
Mean (mg kg^−1^)	15.3863	15.8744	11.5700		
u (mg kg^−1^)	0.3312	0.1124	0.0400		
*u*_rel_	0.0215	0.0071	0.0035	0.0300	0
*u*_com_	0.5809 mg kg^−1^				

### Certified values and traceability

The certified mass fractions of 14 PAHs of BAM-B001 based upon BAM’s in-house study (Table 6) are rounded according to DIN 1333:1992. The values of four additional PAHs (naphthalene, acenaphthylene, acenaphthene, and dibenz[*a,h*]anthracene) were considered as informative due to the reasons specified in the footnotes of Table 6. Based on the calculated *u*_com_ values, expanded uncertainties (*U*) were determined for each PAH congener applying a coverage factor (*k*) of *k* = 2, corresponding to a level of confidence of approximately 95%. The PAH values of the eight REACH-regulated compounds span a relatively wide mass fraction range (0.118 to 2.17 mg/kg). This reflects the natural distribution of the PAHs in this material and allows control of not only the maximum PAH levels for toys (0.5 mg/kg), but also that of other consumer products (1 mg/kg) under REACH.

**Table 6:** Assigned mass fractions of 18 PAH congeners of BAM-B001 (certified and informative values)

Compound	Mass fraction (mg kg^−1^)		Status
	Assigned value	*U*	
Naphthalene	0.09	0.08	Informative^a^
Acenaphthylene	1.6	1.6	Informative^b^
Acenaphthene	0.63	0.28	Informative^c^
Fluorene	1.71	0.22	Certified
Phenanthrene	15.4	1.2	Certified
Anthracene	2.9	1.1	Certified
Fluoranthene	4.3	0.5	Certified
Pyrene	11.4	1.1	Certified
Benz[*a*]anthracene	2.17	0.22	Certified
Chrysene	2.08	0.15	Certified
Benzo[*b*]fluoranthene	0.57	0.05	Certified
Benzo[*k*]fluoranthene	0.213	0.022	Certified
Benzo[*j*]fluoranthene	0.40	0.04	Certified
Benzo[*e*]pyrene	1.21	0.16	Certified
Benzo[*a*]pyrene	1.41	0.10	Certified
Indeno[1,2,3-*cd*]pyrene	0.28	0.06	Certified
Dibenz[*a,h*]anthracene	0.118	0.020	Informative^d^
Benzo[*ghi*]perylene	1.43	0.09	Certified

^a^Large uncertainty of BAM’s naphthalene value; no confirmation by ILCs possible; PAH contents < 0.2 mg/kg are not quantified according to the AfPS GS 2019:01 PAK method

^b^Large uncertainty of BAM’s acenaphthylene value; lacking information from ILC-1

^c^Relatively large uncertainty of BAM’s acenaphthene value; lacking information from ILC-1

^d^PAH contents < 0.2 mg/kg are not quantified according to AfPS GS 2019:01 PAK method; lacking information from ILC-2

All certified values refer to the extractable amounts of the PAH congeners using the extraction procedure of the AfPS GS 2019:01 PAK method and are conventional to this extent. However, different sample preparation procedures were applied so that systematic biases are (at least partially) compensated. To ensure traceability of the extractable amounts as defined above, the gravimetrically prepared certified calibration standard SRM 2260a (NIST) was used for the in-house certification study. Traceability was further demonstrated by SIDA GC–MS measurements.

## Conclusions

In this project, the first CRM for the determination of 18 PAHs in rubber toys was developed, intended to be used to control maximum levels set for PAHs according to REACH regulation and product safety regulation (GS mark, Germany). Production, characterization (homogeneity, stability), and assignment of the certified values of BAM-B001 were performed at BAM in compliance with the internationally accepted procedures laid down in ISO Guide 35. The certified PAH mass fractions of BAM-B001 based upon in-house study using SIDA GC–MS measurements are in good agreement with the results of two interlaboratory comparison studies. Owing to the limited number of matrix CRMs available for analysis of PAH in similar matrices, BAM-B001 is intended for use in the development and validation of new analytical methods and represents an important quality control tool for laboratories to implement and safeguard reliable measurements of PAHs in relevant consumer products, particularly based on the AfPS GS 2019:01 PAK method.

The chemical safety of consumer products is an issue of increasing importance as health standards and public awareness have risen and recycled products are increasingly used as part of the global strategy to strengthen the circular economy. Reliable chemical analyses are necessary to set and control safety standards, but also require CRMs for quality assurance purposes. Such CRMs are urgently needed for consumer product safety in the future. Besides PAHs, CRMs should focus on compounds of emerging concern, e.g., plasticizers, per- and polyfluoroalkyl substances (PFAS), flame retardants, mineral oil saturated/aromatic hydrocarbons (MOSH/MOAH), and potential transformation products.

## Supplementary Information

Below is the link to the electronic supplementary material.

Supplementary file1 (DOCX 48 kb)

## Data Availability

All data and material are available.
