# Supplementary material for: Development of a certified reference material for the determination of polycyclic aromatic hydrocarbons (PAHs) in rubber toy
Source: Anal Bioanal Chem. 2021 Nov 30;414(15):4369–78. doi: 10.1007/s00216-021-03796-5 (PMC9142433; doi:10.1007/s00216-021-03796-5)
Supplement: Supplementary file 1 — Supplementary file1 (DOCX 48 kb) [file 216_2021_3796_MOESM1_ESM.docx]

**Supplementary Information (ESM)**

**Development of a certified reference material for the determination of polycyclic aromatic hydrocarbons (PAHs) in rubber toy**

Thomas Sommerfeld, Christian Jung, Juliane Riedel, Tatjana Mauch, Andreas Sauer, Matthias Koch

All authors:

Bundesanstalt für Materialforschung und -prüfung (BAM), Richard-Willstätter-Straße 11, 12489 Berlin, Germany

**Table S1** Analysis of variance (ANOVA) and estimates for uncertainty contribution due to between-bottle homogeneity in rubber toy reference material BAM-B001

| Compound | Mean^a^ | MS*_between_*^b^ | MS*_within_*^c^ | F*_obs_*^d^ | F*_crit_*^d^ | u*_bb_*^e^ | u*_bb_,_r_*^f^ |
| --- | --- | --- | --- | --- | --- | --- | --- |
|  | mg kg^-1^ | mg^2^ kg^-2^ | mg^2^ kg^-2^ |  |  | mg kg^-1^ |  |
| Naphthalene | 0*.*0639 | 0*.*00001 | 0*.*00001 | 0*.*8776 | 2*.*0666 | 0 | 0 |
| Acenaphthylene | 0*.*9647 | 0*.*00399 | 0*.*00707 | 0*.*5642 | 2*.*0666 | 0 | 0 |
| Acenaphthene | 0*.*7890 | 0*.*00226 | 0*.*00263 | 0*.*8600 | 2*.*0666 | 0 | 0 |
| Fluorene | 1*.*6892 | 0*.*00948 | 0*.*00381 | 2*.*4846 | 2*.*0666 | 0*.*0376 | 0*.*0223 |
| Phenanthrene | 15*.*8744 | 0*.*15350 | 0*.*10296 | 1*.*4909 | 2*.*0666 | 0*.*1124 | 0*.*0071 |
| Anthracene | 2*.*5201 | 0*.*01065 | 0*.*01071 | 0*.*9947 | 2*.*0666 | 0 | 0 |
| Fluoranthene | 4*.*5275 | 0*.*01136 | 0*.*00757 | 1*.*5009 | 2*.*0666 | 0*.*0309 | 0*.*0068 |
| Pyrene | 11*.*9476 | 0*.*06998 | 0*.*05639 | 1*.*2409 | 2*.*0666 | 0*.*0583 | 0*.*0049 |
| Benz[*a*]anthracene | 2*.*3173 | 0*.*00181 | 0*.*00135 | 1*.*3381 | 2*.*0666 | 0*.*0107 | 0*.*0046 |
| Chrysene | 2*.*1127 | 0*.*00263 | 0*.*00181 | 1*.*4580 | 2*.*0666 | 0*.*0144 | 0*.*0068 |
| Benzo[*b*]fluoranthene | 0*.*5988 | 0*.*00013 | 0*.*00013 | 1*.*0027 | 2*.*0666 | 0.0003 | 0.0005 |
| Benzo[*k*]fluoranthene | 0*.*2146 | 0*.*00019 | 0*.*00033 | 0*.*5924 | 2*.*0666 | 0 | 0 |
| Benzo[*j*]fluoranthene | 0*.*3954 | 0*.*00010 | 0*.*00016 | 0*.*5888 | 2*.*0666 | 0 | 0 |
| Benzo[*e*]pyrene | 1*.*3079 | 0*.*00035 | 0*.*00061 | 0*.*5779 | 2*.*0666 | 0 | 0 |
| Benzo[*a*]pyrene | 1*.*4237 | 0*.*00037 | 0*.*00074 | 0*.*4980 | 2*.*0666 | 0 | 0 |
| Indeno[1,2,3-*cd*]pyrene | 0*.*2728 | 0*.*00018 | 0*.*00022 | 0*.*8407 | 2*.*0666 | 0 | 0 |
| Dibenz[*a,h*]anthracenene | 0*.*1355 | 0*.*00041 | 0*.*00041 | 0*.*9903 | 2*.*0666 | 0 | 0 |
| Benzo[*ghi*]perylene | 1*.*4340 | 0*.*00072 | 0*.*00086 | 0*.*8402 | 2*.*0666 | 0 | 0 |

^a^ Mean of the homogeneity study (= mean of bottle means)

^b^ Mean of squared deviation between units (from 1-way ANOVA)

^c^ Mean of squared deviation within units (from 1-way ANOVA)

^d^ Observed F-value (= MS*_between_*/MS*_within_*) and critical F-value (from F-value table; significance level α, α=0.05)

^e^ Standard uncertainty between the units: Estimate of inhomogeneity contribution to the total uncertainty

^f^ Relative standard uncertainty between the units (u*_bb_* / mean of homogeneity study)

**Table S2** Mean recoveries (± standard deviation) of PAHs in rubber toy (BAM-B001) after 12 months at different storage temperatures

| Compound | Recovery (%) | | | | Remark |
| --- | --- | --- | --- | --- | --- |
|  | +4 °C | +23 °C | +40 °C | +60 °C |  |
| Naphthalene | 100.7 ± 6.8 | 98.4 ± 6.3 | 108.7 ± 8.5 | --- | Long-term study |
| Acenaphthylene | 109.2 ± 5.5 | 108.8 ± 4.3 | 99.4 ± 4.9 | --- | Long-term study |
| Acenaphthene | 82.5 ± 43.3 | 100.7 ± 33.9 | 105.6 ± 22.8 | --- | Long-term study |
| Fluorene | 104.4 ± 2.1 | 105.1 ± 1.9 | 104.0 ± 2.6 | --- | Long-term study |
| Phenanthrene | 100.4 ± 0.9 | 100.5 ± 0.8 | 99.8 ± 1.2 | --- | Long-term study |
| Anthracene | 102.2 ± 1.2 | 101.7 ± 1.2 | 100.0 ± 0.9 | --- | Long-term study |
| Fluoranthene | 100.4 ± 0.8 | 100.7 ± 0.8 | 100.4 ± 0.5 | --- | Long-term study |
| Pyrene | 100.0 ± 0.7 | 100.4 ± 0.8 | 99.7 ± 0.5 | --- | Long-term study |
| Benz[*a*]anthracene | 99.1 ± 2.2 | 99.9 ± 1.6 | 99.3 ± 1.8 | 99.3 ± 1.5 | Short- and long-term |
| Chrysene | 99.5 ± 1.7 | 100.4 ± 2.3 | 99.3 ± 1.8 | 98.7 ± 3.0 | Short- and long-term |
| Benzo[*b*]fluoranthene | 98.6 ± 2.0 | 98.8 ± 1.2 | 98.5 ± 2.0 | 98.7 ± 2.7 | Short- and long-term |
| Benzo[*k*]fluoranthene | 100.1 ± 3.1 | 99.3 ± 3.0 | 100.0 ± 3.8 | 99.0 ± 3.6 | Short- and long-term |
| Benzo[*j*]fluoranthene | 104.2 ± 2.9 | 102.7 ± 3.7 | 102.6 ± 2.1 | 101.3 ± 1.9 | Short- and long-term |
| Benzo[*e*]pyrene | 99.5 ± 1.8 | 99.9 ± 1.6 | 99.3 ± 1.7 | 99.0 ± 1.8 | Short- and long-term |
| Benzo[*a*]pyrene | 99.8 ± 1.9 | 101.2 ± 2.0 | 99.7 ± 1.8 | 100.9 ± 3.6 | Short- and long-term |
| Indeno[1,2,3-*cd*]pyrene | 100.6 ± 6.7 | 103.3 ± 8.0 | 102.2 ± 8.9 | 107.0 ± 8.6 | Short- and long-term |
| Dibenz[*a,h*]anthracene | 96.5 ± 5.0 | 96.8 ± 5.0 | 97.0 ± 6.2 | 99.1 ± 4.0 | Short- and long-term |
| Benzo[*ghi*]perylene | 100.4 ± 2.1 | 100.7 ± 2.1 | 100.7 ± 1.4 | 99.2 ± 1.7 | Short- and long-term |

**Table S3** Three data sets for the in-house certification study of BAM-B001 (values in mg kg^-1^)

| Compound | Data set 1 | | Data set 2 | | Data set 3 | |
| --- | --- | --- | --- | --- | --- | --- |
|  | Workplace A | | Workplace B | | Homogeneity study * | |
|  | Mean | SD | Mean | SD | Mean | SD |
| Naphthalene | 0.043 | 0.016 | 0.169 | 0.056 | 0*.*0639 | 0.0031 |
| Acenaphthylene | 0.784 | 0.067 | 3.161 | 0.893 | 0*.*9647 | 0.0797 |
| Acenaphthene | 0.755 | 0.091 | 0.359 | 0.020 | 0*.*7890 | 0.0504 |
| Fluorene | 1.580 | 0.034 | 1.874 | 0.085 | 1*.*6892 | 0.0717 |
| Phenanthrene | 15.530 | 0.164 | 14.754 | 0.302 | 15*.*8744 | 0.3388 |
| Anthracene | 2.267 | 0.032 | 3.894 | 0.294 | 2*.*5201 | 0.1034 |
| Fluoranthene | 4.289 | 0.046 | 4.004 | 0.054 | 4*.*5275 | 0.0920 |
| Pyrene | 11.463 | 0.080 | 10.654 | 0.182 | 11*.*9476 | 0.2441 |
| Benz[*a*]anthracene | 2.183 | 0.014 | 2.022 | 0.053 | 2*.*3173 | 0.0382 |
| Chrysene | 2.047 | 0.022 | 2.083 | 0.104 | 2*.*1127 | 0.0447 |
| Benzo[*b*]fluoranthene | 0.569 | 0.008 | 0.554 | 0.012 | 0*.*5988 | 0.0114 |
| Benzo[*k*]fluoranthene | 0.198 | 0.020 | 0.227 | 0.005 | 0*.*2146 | 0.0172 |
| Benzo[*j*]fluoranthene | 0.388 | 0.016 | 0.417 | 0.009 | 0*.*3954 | 0.0122 |
| Benzo[*e*]pyrene | 1.238 | 0.012 | 1.077 | 0.029 | 1*.*3079 | 0.0234 |
| Benzo[*a*]pyrene | 1.423 | 0.010 | 1.370 | 0.021 | 1*.*4237 | 0.0256 |
| Indeno[1,2,3-*cd*]pyrene | 0.243 | 0.011 | 0.334 | 0.006 | 0*.*2728 | 0.0145 |
| Dibenz[*a,h*]anthracene | 0.112 | 0.011 | 0.107 | 0.004 | 0*.*1355 | 0.0203 |
| Benzo[*ghi*]perylene | 1.416 | 0.022 | 1.455 | 0.037 | 1*.*4340 | 0.0288 |

*) The homogeneity study was carried out by operator A using method A

Mean mean of 5 bottle means (for data sets A and B); mean of 12 bottle means (for data set 3)

SD standard deviation of 25 single values (for data sets A and B); standard deviation of 48 single values (for data set 3)

**Table S4** Uncertainty contributions for the calculation of the combined uncertainty (u_com_) of benz[*a*]anthracene in BAM-B001

| Parameter | Characterization | Homogeneity | Purity | Handling | Stability |
| --- | --- | --- | --- | --- | --- |
| Mean (mg kg^-1^) | 2.1740 | 2.3173 | 4.4150 |  |  |
| u (mg kg^-1^) | 0.0853 | 0.0107 | 0.0260 |  |  |
| u*_rel_* | 0.0393 | 0.0046 | 0.0059 | 0.0300 | 0 |
| u*_com_* | 0.1086 mg kg^-1^ | | | | |

**Table S5** Uncertainty contributions for the calculation of the combined uncertainty (u_com_) of benzo[*k*]fluoranthene in BAM-B001

| Parameter | Characterization | Homogeneity | Purity | Handling | Stability |
| --- | --- | --- | --- | --- | --- |
| Mean (mg kg^-1^) | 0.2133 | 0.2146 | 3.4440 |  |  |
| u (mg kg^-1^) | 0.0085 | 0.0000 | 0.0120 |  |  |
| u*_rel_* | 0.0397 | 0.0000 | 0.0035 | 0.0300 | 0 |
| u*_com_* | 0.0106 mg kg^-1^ | | | | |

**Table S6** Uncertainty contributions for the calculation of the combined uncertainty (u_com_) of benzo[*a*]pyrene in BAM-B001

| Parameter | Characterization | Homogeneity | Purity | Handling | Stability |
| --- | --- | --- | --- | --- | --- |
| Mean (mg kg^-1^) | 1.4056 | 1.4237 | 4.7100 |  |  |
| u (mg kg^-1^) | 0.0178 | 0.0000 | 0.0567 |  |  |
| u*_rel_* | 0.0126 | 0.0000 | 0.0120 | 0.0300 | 0 |
| u*_com_* | 0.0488 mg kg^-1^ | | | | |

**Table S7** Combined uncertainties of analyzed PAHs in BAM-B001

| Compound | u*_com_* (mg kg^-1^) | Compound | u*_com_* (mg kg^-1^) |
| --- | --- | --- | --- |
| Naphthalene | 0.0390 | Chrysene | 0.0712 |
| Acenaphthylene | 0.7660 | Benzo[*b*]fluoranthene | 0.0219 |
| Acenaphthene | 0.1396 | Benzo[*k*]fluoranthene | 0.0106 |
| Fluorene | 0.1089 | Benzo[*j*]fluoranthene | 0.0156 |
| Phenanthrene | 0.5809 | Benzo[*e*]pyrene | 0.0774 |
| Anthracene | 0.5130 | Benzo[*a*]pyrene | 0.0488 |
| Fluoranthene | 0.2010 | Indeno[1,2,3-*cd*]pyrene | 0.0281 |
| Pyrene | 0.5125 | Dibenz[*a,h*]anthracene | 0.0096 |
| Benz[*a*]anthracene | 0.1086 | Benzo[*ghi*]perylene | 0.0449 |
